# Patients’ Intention to Adopt Fintech Services: A Study on Bangladesh Healthcare Sector

**DOI:** 10.3390/ijerph192215302

**Published:** 2022-11-19

**Authors:** Md. Sharif Hassan, Md. Aminul Islam, Farid Ahammad Sobhani, Md. Maruf Hassan, Md. Arif Hassan

**Affiliations:** 1 Department of Business, Faculty of Business and Communication, Universiti Malaysia Perlis, Kangar 01000, Malaysia; 2 Department of Business Administration, University of Asia Pacific, Dhaka 1205, Bangladesh; 3 Center of Excellence for Social Innovation and Sustainability, Universiti Malaysia Perlis, Kangar 01000, Malaysia; 4 School of Business and Economics, United International University, Dhaka 1212, Bangladesh; 5 Department of Software Engineering, Daffodil International University, Dhaka 1341, Bangladesh; 6 Department of Business Administration, Faculty of Business and Entrepreneurship, Daffodil International University, Dhaka 1341, Bangladesh

**Keywords:** fintech services, healthcare, perceived ease of use, social influence, facilitating conditions, personal innovativeness, perceived trust

## Abstract

Advancement in technology has facilitated the shift toward new financial services. Numerous industries have undergone a digital transformation because of the expansion of cashless payment systems and other cutting-edge technologies. This study aimed to identify the factors that stimulate the patient’s intention to adopt fintech services in the Bangladesh healthcare sector. To facilitate the study, data were collected through survey questionnaires from different hospitals and diagnostic centers patients. A total of 279 patients responded to the survey. The study employed structural equation modelling to analyze the data using SMART PLS 3.2.9. The results revealed that a significant relationship exists between perceived ease of use, social influence, facilitating conditions, personal innovativeness, and perceived trust in fintech services, and the adoption intention of the patients. The results of the study are beneficial to the healthcare sector and fintech companies who wish to make necessary arrangements to advance the growth of cashless fintech-based transactions.

## 1. Introduction

Digital transformation has changed the way organizations are now operating in different sectors. Today, people of all ages use technological products and services to save their time, money, and energy. Financial technology (fintech) is an emerging technology service introduced by financial companies and banks and is now very popular among the users of the financial and non-financial industries. Fintech is the combination of traditional financial services and technology. According to Kim et al. [1], fintech is a service that provides mobile-based IT technological services to enrich the financial system’s efficiency. It can also be defined as the combination of business models and technology by organizations to enable, enhance, and disrupt financial services.

Investors around the world are significantly investing in fintech firms to innovate new products and services so that the financial service process can be easier for customers. The investment in global fintech companies rose from USD 9 billion to 210.1 billion in 2021 [2]. According to a study conducted in 27 emerging markets, the knowledge of fintech services has risen among people globally compared to 2015; 96% of people are aware of at least one fintech service and 64% of people already adopted the fintech services [3].

Banking, marketing, and manufacturing firms have been the early adopters of fintech. The use of fintech services in the health care sector is not as promising as in other sectors, and the adoption is quite slow. Nevertheless, the healthcare sector is now offering a different kind of fintech services. Globally, hospitals are now offering digital payment services. To promote digital payment, discounts are even given to attract patients and customers. The emergence of the digital payment system is enabling patients to securely pay their hospital, medicine, and doctor bills using different mobile-based fintech services [4]. 

Bangladesh healthcare includes hospitals, diagnostic centers, tele-medicines, clinics, and medical devices and equipment. According to BIDA, in 2018 the size of this sector was around USD 6.76 billion, with a CAGR of 10.3% [5]. Currently, the sector is flourishing and has significant potential. However, patients in this sector have to bear difficulties in making financial transactions with the healthcare facilities if they follow the traditional payment approach. The payment process has always been a challenge in the healthcare industry and a place where numerous human errors, such as lost receipts and problems with payment verification, occur. In addition, the patients need to stand in line and pay the requested amount physically at the cash counters, which is difficult for critical patients. Treatment only starts after the payment is made by the patients. Protecting patient data is one of the biggest issues facing the healthcare industry. Patient data frequently contains personal information that should be protected from falling into the wrong hands. According to Becker’s Hospital Review, the payment-related problems that are commonly faced by patients in healthcare facilities are a lack of price transparency of medical expenses, a complicated billing process, and a lack of automated billing [6]. These difficulties and problems arise for the patients while receiving treatment from the healthcare facilities. Patients can minimize the problems and difficulties that they face during the payment process in health facilities by adopting fintech services offered by healthcare facilities. Fintech services can be used to make paying for healthcare easier, streamline the billing and payment process for the patients, change the management of patient data, and develop personalized programs for the patients [7]. However, the usage of fintech services in the healthcare sector is quite new to the patients and the health facilities. Both hospitals and patients tend to use traditional financial services, which increases the administrative cost of the hospitals and the risk to patients of carrying cash. Recently, private hospitals located in the major cities of Bangladesh have introduced fintech payment systems to collect payments for hospital bills, doctor bills, medicines, and other services. Some hospitals have even developed mobile apps for patients to offer smooth fintech services. According to Surf, people do not trust fintech services due to low awareness, uncertainty about data privacy, and the fear of the unknown [8]. Nonetheless, fintech services can improve the patient’s experience at healthcare facilities and smooth the payment-receiving process for the healthcare facilities. Thus, there is a significant need to increase the adoption of fintech services among patients, which would help to reduce the risk and difficulties of using traditional financial services, and broaden financial access to reduce complex transaction costs and expedite payment processes. 

Prominent theories and models, such as TAM, UTAUT/UTAUT2, and Diffusion of Innovation theory, have been used significantly in technology adoption research [9,10,11]. This study aimed to identify the factors that are impacting the patient’s intention to use fintech services in the healthcare industry of Bangladesh. The study utilized variables from different models and proposed a research framework considering the existing problems faced by the patients in Bangladesh’s healthcare sector, which can explain the patients’ intention to adopt fintech services. Scant studies currently exist on global fintech service adoption in the healthcare industry. This study will contribute to the literature on fintech service adoption in the healthcare industry and help the healthcare stakeholders to understand the drivers behind using fintech services by the patients.

The paper is organized in the following sections: literature review, methodology, findings and discussion, and conclusion.

## 2. Literature Review and Hypothesis Development

Due to the emergence of COVID-19, fintech services’ role in society has become more crucial than ever [12]. Globally, the reason for the adoption of fintech services is that people can access the services anytime from anywhere. In addition, these services save time, money, and energy. Several researchers have conducted a study on fintech adoption. Singh et al. conducted a study on fintech adoption and contributed to the technology acceptance literature [13]. In addition, Ryu [14] conducted a study on fintech adoption of early adopters and late adopters. Setiwana et al. performed a study on fintech adoption in Indonesia among fintech users [15]. A study was also conducted in Australia on open banking (fintech) services adoption [16]. Moreover, Bureshaid et al. [17] analyzed fintech adoption in the banking industry. In most of the studies, the adoption of fintech took place in the banking, insurance, and other related financial sectors. However, research on fintech services adoption in the healthcare sector is scarce.

Perceived ease of use is considered one of the potential determinants of technology adoption. This can be defined as the ease of using a system that is free of effort [18]. This independent variable was used in the TAM model developed by Davis. If the technology or system is simple to understand and easily accessible there is more chance that people will intend to adopt that service. Rahmi et al. [19] utilized perceived ease of use (PEOU) to analyze the adoption of e-learning systems and found that PEOU has a positive impact. Nangin et al. used PEOU as the antecedent of trust that influences fintech adoption; the results revealed that PEOU impacts trust and trust positively impacts fintech adoption [20]. In a study conducted on Taiwanese consumers, PEOU was found to have a positive impact on e-purchase intention [21]. Similar results were found in a study using online travel agencies [22].

The importance of others (family, friends) in convincing a person to utilize a certain technology is known as a social influence [23]. It is one of the independent predictors used in the UTAUT and UTAUT2 model by Venketesh et al. This is also a common independent variable in technology adoption, whether in the context of a business or a customer. According to the research on Malaysian banks, social influence has a positive impact on the adoption of blockchain technologies [24]. Similar results were found for mobile wallet adoption [25]. However, in a study on adopting mobile banking, it was discovered that social influence does not affect adoption intentions [26].

The available resources and support to undertake a behavior that is perceived by the customer are referred to as facilitating conditions [27]. This variable was introduced in the UTAUT model [28]. Customers’ ease of use of technology in purchasing items and services is aided by facilitating conditions [29]. In research on developing Asian economics, it was discovered that facilitating conditions had a considerable positive impact on the adoption of Internet banking [30]. Furthermore, in the case of mobile wallet usage in Indonesia, facilitating conditions are a key predictor [31]. However, no impact was found in several studies [32,33]. On the contrary, facilitating conditions were found to have to negative impact on electric vehicle adoption in Malaysia [34].

In the case of technological acceptance, personal innovativeness (PI) is a critical aspect that determines consumer attitude and intention to buy, buying decisions, consumer satisfaction, and loyalty [35]. Personal innovativeness refers to a person’s propensity to experiment with new technology [36]. Continuous PI usage was found to be a powerful predictor of early m-commerce adoption [37]. A similar result was seen in the case of students’ intentions to use cloud classrooms [38]. Furthermore, in a study conducted in Thailand to determine whether people intend to use the QR payment method, PI was found to be a strong positive indication [39].

Trust is a crucial element in the context of fintech services adoption because of the lack of a personal touch, the high degree of unpredictability, and the nature of the Internet [40]. Kim et al. [41] used trust in the extended valence framework as one of the core components to measure behavioral intention to use technology. Alalwan et al. [26] included trust in the UTAUT2 model and discovered that trust has a significant positive impact on behavioral intention relating to mobile banking acceptance. According to Stewart and Jürjens [42], consumer trust has a strong positive influence on fintech innovation adoption. A study on actual health insurance system utilization reached the same conclusion [43].

**Figure 1:**
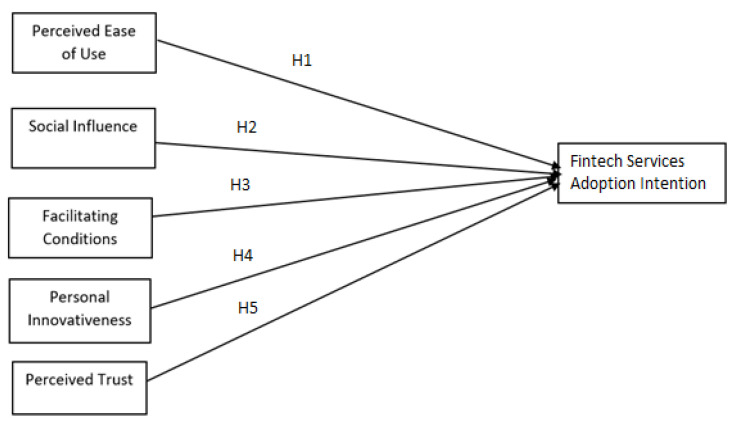
Research framework.

From the above discussion, it is evident that no studies have been conducted on patients’ intention to adopt fintech services in the Bangladesh healthcare sector. This study will minimize the gap in the literature and determine the factors that influence patients’ intention to adopt fintech services. The study proposes the following research framework showed in Figure 1.

### 2.1. Behavioral Intention to Adopt Fintech Services

Behavioral intention can be defined as the probability of a person performing some behavior [44]. Behavioral intentions are psychological drivers that measure the amount of effort a person is prepared to expend to carry out a behavior [45]. In this study, behavioral intention to use fintech services was used as the dependent variable. Earlier studies also used behavioral intention as the dependent variable [46,47].

### 2.2. Perceived Ease of Use

Perceived ease of use refers to the ease of using a system. Perceived ease of use was found to have a positive influence on repurchase intention in the Indonesian e-commerce industry [48]. Similar results were found in the case of instructors’ intentions to use a learning management system [49]. However, no effect was found in a study conducted on students’ intention to use mobile devices in language learning [50]. Based on the above discussion this study proposes the following hypothesis:

**H1.**  *Perceived ease of use (PEOU) is positively related to behavioral intention to use fintech services (BI).*

### 2.3. Social Influence

Social influence is one of the common indicators of an intention to use. It refers to the influence of family and friends of a person to use a particular system. Social influence showed positive influence in the case of Internet banking adoption intention [51]. Similar results were found in a study on fintech adoption conducted in Jordan [46]. This study proposes the following hypothesis:

**H2:**  *Social influence (SI) is positively related to behavioral intention to use fintech services (BI).*

### 2.4. Facilitating Conditions

Facilitating conditions are the available resources a person has to perform any particular behavior. A study of Chinese live e-commerce shopping intention facilitating conditions showed a positive relationship towards behavioral intention [52]. The same result was found in the case of m-commerce adoption intention [53]. Based on the discussion, this study proposes the following hypothesis:

**H3:**  *Facilitating conditions (FCs) are positively related to behavioral intention to use fintech services (BI).*

### 2.5. Personal Innovativeness

Personal innovativeness is considered as the propensity of an individual to try something different from another person. Personal innovativeness positively influenced information technology adoption intention [54]. In addition, in a study of QR payment adoption intention studyl innovativeness showed a positive impact [27]. The study proposes the following hypothesis based on the discussion:

**H4:**  *Personal innovativeness (PI) is positively related to behavioral intention to use fintech services (BI).*

### 2.6. Perceived Trust

Trust is the confidence a person has in the capacity of an object to behave consistently, forcefully, and dependably in a certain situation [55]. According to a study, customer desire to use technology is positively impacted by trust [56]. In a study conducted on the Indonesian young generation, perceived trust showed a positive influence on behavioral intention [57]. The study proposes the following hypothesis.

**H5:**  *Perceived trust (PT) is positively related to behavioral intention to use fintech services (BI).*

## 3. Methodology

This study used a deductive approach. A survey questionnaire was developed to gather the respondents’ observations. The observation confirmed the acceptance or rejection of the hypothesis. The explanatory research design was employed in the study’s execution. This study produced new data regarding patients’ intention to use fintech services in the healthcare sector.

### 3.1. Sample and Data Collection

The study was mainly focused on the healthcare sector of Bangladesh, and followed non-probability purposive sampling. Barclay et al. [58] mentioned that the minimum sample size should be “ten times the biggest number of formative indicators used to measure one construct” or “ten times the largest number of inner model paths directed at a single construct in the inner model”, depending on which is higher. The study included five independent predictors: perceived ease of use, social influence, facilitating conditions, personal innovativeness, and perceived trust. The measurement constructs of the variables were adapted from previous studies. Three measurement constructs of perceived ease of use were adapted from a study on fintech adoption in Indonesia [15] and one construct from the TAM model developed by Davis [18]. The constructs for social influence, facilitating conditions, and behavioral intention were adapted from technology adoption studies [46,59]. In addition, measurement instruments of personal innovativeness were adapted from earlier literature on fintech services [60,61]. Measurement instruments for perceived trust were adapted from a study conducted on technology adoption [62]. In total, there were 19 measurement items. According to the rule of Barclay et al., the minimum sample size for the study should be 190. However, this study maintained the minimum requirement and finally used 279 samples for the analysis. The study used a seven-point Likert scale to evaluate the measurement items, as used in earlier studies [63], because it provides the highest variance [64]. The survey questionnaire was distributed to patients at 5 hospitals and diagnostic centers located in Dhaka. The measurement items are shown in Appendix A of the paper. Collected data were inserted into an Excel file for exporting to SMARTPLS software.

### 3.2. Data Analysis

To analyze the data, SMART PLS 3.2.9 software was used because this software is best suited for small data samples [65]. To facilitate the study, structural equation modeling (SEM) was utilized. The study utilized PLS-SEM because, to identify key driver constructs, PLS-SEM is recommended [66]. According to Hair et al. [67], PLS-SEM consists of a measurement model and a structural model, where the measurement model is used to assess the validity and reliability of the constructs, and the structural model assesses the explanatory power, multicollinearity issues, and significance of path coefficients.

## 4. Findings and Analysis

### 4.1. Demographic Profile

The demographic profile of the study is given in Table 1.

The survey questionnaire started with questions regarding the awareness of the patients of fintech services provided by the healthcare sector. About 92.8% responded that they are aware of the fintech services provided by the healthcare sector. The shares of male and female respondents for the study were 62.1% and 37.9%. Most of the respondents were aged between 26 and 45, which accounted for about 69.2%. In addition, about 75.3% of respondents were employed, working at different firms and organizations.

### 4.2. Measurement Model

The major task of the measurement model is to verify the reliability and validity of the instruments used in the research framework. The first step of the measurement model is to verify the reliability of indicator loadings of the construct. According to Hair et al. [67], the suggested indicator loadings should be more than 0.708. Then, internal consistency reliability needs to be verified; two indicators were used, namely, Cronbach’s alpha and composite reliability. The value should be more than 0.70 [65]. Table 2 and Table 3 contain the outer loading values, including reliability and validity scores. According to results shown in Table 2 and Table 3, the outer-loading value, reliability, and validity scores meet the requirements of the measurement model.

After confirming the reliability, the next step was to verify the convergent validity. The average variance extracted (AVE) was used as the indicator to check the convergent validity of the constructs. The expected value of the AVE is 0.50 [67]. Verifying the discriminant validity is the last step. The Fornell–Larcker criterion, heterotrait–monotrait (HTMT) ratio, and cross-loading are three separate methods used to test the discriminant validity. Whether the square root of the AVE of a certain construct is bigger than other constructs is determined using the Fornell–Lacker criterion [68]. For the cross-loading criteria, a construct’s parent construct should contain higher loadings for cross-loading than other constructs. Moreover, the threshold value for conceptually connected components in structural models, according to Henseler et al. [69], should be 0.90, whereas the threshold for conceptually independent constructs should be 0.85. Insufficient discriminant validity is indicated by a higher value. The results for the Fornell–Lacker values and HTMT scores are given in Table 4 and Table 5. The results indicate that the constructs met the requirements of discriminant validity.

### 4.3. Structural Model Assessment

The structural model employed in the study was assessed utilizing the explanatory power and statistical significance of the path coefficients. It is important to assess the constructs’ multicollinearity before evaluating the structural model. The VIF values should be less than 5 to demonstrate no multicollinearity exists [65]. Table 6 shows that all VIF values satisfy the requirement.

**Figure 2:**
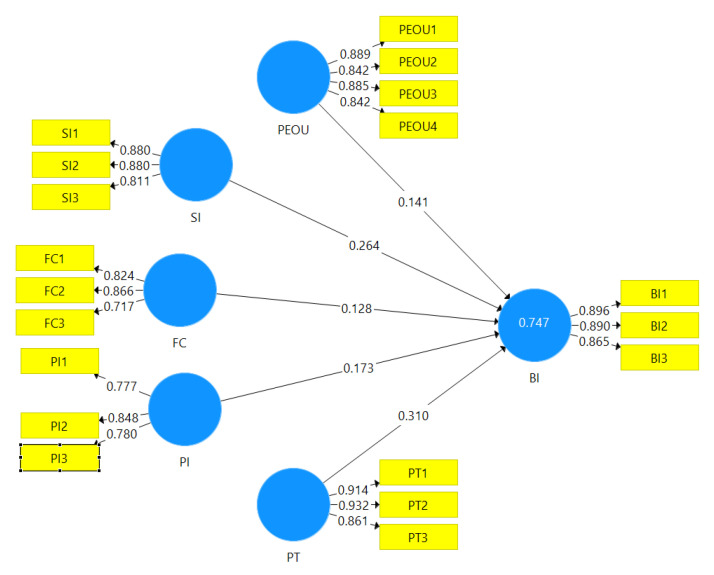
R^2^ Value. Source: SMART PLS Analysis Result.

Figure 2 indicates that the coefficient of determination (R^2^) value of the model is 0.747, which is substantial. This means the explanatory power of the model is 74.7%. The model can explain 74.7% of healthcare sector patients’ behavioral intention to adopting fintech services.

The hypothesis of a study is accepted or rejected based on the path coefficients’ *p*-value. The criterion for accepting a hypothesis is that the *p*-value of the hypothesis should be less than 0.05 [46]. According to Table 7, all the hypotheses of the study are accepted because the *p*-values of all the hypothesis are less than 0.05. Thus, H1, H2, H3, H4, and H5 are accepted.

### 4.4. Discussion

The healthcare sector is an emerging sector in Bangladesh. According to the World Bank, the expenditure of Bangladesh’s healthcare sector accounted for 2.48% of GDP in 2019 [70]. The average expenditure per population increased by 9.43% compared to 2018 [71]. This indicates that the average spending of the people of the country is increasing. Moreover, due to the increase in the usage of smartphones and Internet, fintech services such as digital payments have also become popular in the country. The number of mobile phone users has substantially increased. Bangladesh had 178.61 million mobile phone subscribers as of August 2021. Additionally, Bangladesh has a 31.5% Internet user penetration rate. According to Statista, the volume of fintech transactions only in digital payments increased by 31.42% in 2022 compared to 2020 [72]. This clearly shows the growth of the usage of fintech services in Bangladesh.

The purpose of the study was to identify the factors that are impacting the patients’ intentions to adopt fintech services in the healthcare sector of Bangladesh. The study proposed a research framework comprising five independent variables, namely, perceived ease of use, social influence, facilitating conditions, personal innovativeness, and perceived trust, based on the technology adoption literature.

The results of the study indicate that perceived ease of use and facilitating conditions have a positive influence on the fintech adoption intention of the patients. The findings of the study are similar to those of previous studies [21,49,52]. The user interface that is implemented by banks for their digital fintech payment apps is user-friendly. People can choose the language and, in some cases, the apps can be used without the Internet, which allows non-smartphone or Internet users to make fintech transactions. Banks and hospitals operate 24 h contact-center services so that, if patients experience any trouble in making payments, they can easily contact the call center for any digital payment-related issues. The ease of use and availability of the technology are directly prompting the patients to use fintech services in the healthcare sector. In addition, the study also revealed that social influence, personal innovativeness, and perceived trust are significant positive indicators that influence patients’ intentions to adopt fintech services. Similar results were found in earlier studies [43,51,54]. Family and friends play a critical role in Bangladesh culture. People are fond of their family and friends. They tend to follow suggestions made by people close to them. In addition, personal level creativity and risk-taking nature also influence an individual to try new digital and online services. Most importantly, the structural dependency of the fintech service, operator reputation, and personal propensity to trust impact a user’s intention to adopt fintech services in the healthcare sector of Bangladesh. Thus, the fintech firms and banks should focus on the user-friendliness of the apps, create more awareness about fintech through extensive marketing, and collaborate with new business partners to increase the growth in fintech services in Bangladesh. Moreover, the healthcare sector should encourage patients to use digital channels for payments and other related services, which will eventually result in savings in the cash management of the hospitals and diagnostic centers and reduce the patient’s risk of carrying cash. The summary of hypotheses results is given below in Table 8.

## 5. Conclusions

The era of the fourth industrial revolution has prompted organizations to adopt new technologies to perform their businesses. There has been tremendous change in the way people are now performing transactions to purchase any product or service. The emergence of fintech services has made it easier for companies and consumers to easily conduct cashless transactions. This change is also visible in different sectors of Bangladesh. There has been significant growth in fintech transactions in the past few years. This study aimed to identify the factors that influence the patient’s intention to adopt fintech services in the healthcare sector of Bangladesh. The study revealed that perceived ease of use, social influence, facilitating conditions, personal innovativeness, and perceived trust influence the intention of healthcare sector patients to adopt fintech services. The user-friendly interface of the apps, 24 h customer care service, and payment assistance manuals in hospital and diagnostic center premises influence the patients to use fintech services. In addition, individual-level innovativeness is an important influence on whether a person intends to use new services. Moreover, suggestions from family and friends eventually motivate people to use new fintech services. Furthermore, the perceived trust regarding the service also influences patients to use fintech services, which is based on the perception of fintech firms’ reputation and structural assurance. The acceptance of fintech services in the healthcare sector will boost the savings for the healthcare organizations in cash management and protect the patients from the risk of carrying cash.

### 5.1. Managerial Implications

The results of the study will be beneficial for both the healthcare sector and fintech firms. Based on these results, fintech firms need to work on the interface of the app to make it easier, so that people of different ages and education qualifications can use the apps. In addition, they need to create more awareness about the services through marketing to reach more potential customers. They also need to make sure the transaction process is transparent, and that people can perform secure transactions. The hospitals and diagnostic centers should promote and enable fintech payments for patients, which will reduce the cost and risk of cash management.

### 5.2. Limitations and Future Research

The study has a few limitations. The sample was only based on Dhaka city hospitals and diagnostic centers. This restricts the generalizability of the research findings across various Bangladeshi demographic classes and geographic regions. In addition, only five variables were utilized from different technology adoption models. Future studies can introduce new variables such as perceived security, risk, and user resistance, in addition to using trust as a moderating variable.

## Figures and Tables

**Table 1:** Demographic profile of the study.

Demographic Variable	Frequency	Percentage
Are you aware of the fintech services provided by the healthcare sector?		
Yes	259	92.8
No	20	7.2
Total	279	100.0
Gender		
Male	173	62.1
Female	106	37.9
Total	279	100.0
Age		
16–25	31	11.1
26–35	125	44.8
36–45	68	24.4
46–55	43	15.4
55 above	12	4.3
Total	279	100.0
Employment Status		
Employed	210	75.3
Un-Employed	32	11.5
Self Employed	22	7.9
Student	8	2.9
Others	7	2.4
Total	279	100.0

Source: SMART PLS Analysis Result.

**Table 2:** Outer loading values.

	BI	FC	PEOU	PI	PT	SI
BI1	0.896					
BI2	0.890					
BI3	0.865					
FC1		0.824				
FC2		0.866				
FC3		0.717				
PEOU1			0.889			
PEOU2			0.842			
PEOU3			0.885			
PEOU4			0.842			
PI1				0.777		
PI3				0.848		
PI4				0.780		
PT1					0.914	
PT2					0.932	
PT3					0.861	
SI1						0.880
SI2						0.880
SI3						0.811

Source: SMART PLS Analysis Result.

**Table 3:** Reliability and convergent validity scores.

	Cronbach’s Alpha	Composite Reliability	Average Variance Extracted (AVE)
BI	0.860	0.915	0.782
FC	0.729	0.846	0.648
PEOU	0.887	0.922	0.748
PI	0.722	0.844	0.644
PT	0.887	0.930	0.815
SI	0.819	0.893	0.735

Source: SMART PLS Analysis Result.

**Table 4:** Fornell–Lacker criteria.

	BI	FC	PEOU	PI	PT	SI
BI	0.884					
FC	0.706	0.805				
PEOU	0.709	0.712	0.865			
PI	0.706	0.641	0.633	0.802		
PT	0.766	0.626	0.637	0.642	0.903	
SI	0.752	0.660	0.647	0.620	0.666	0.857

Source: SMART PLS Analysis Result.

**Table 5:** HTMT scores.

	BI	FC	PEOU	PI	PT	SI
BI						
FC	0.877					
PEOU	0.810	0.874				
PI	0.895	0.859	0.788			
PT	0.870	0.756	0.711	0.796		
SI	0.896	0.853	0.760	0.805	0.777	

Source: SMART PLS Analysis Result.

**Table 6:** VIF scores.

	VIF
BI1	2.316
BI2	2.345
BI3	1.976
FC1	1.463
FC2	1.646
FC3	1.347
PEOU1	2.726
PEOU2	2.058
PEOU3	2.761
PEOU4	2.081
PI1	1.345
PI3	1.641
PI4	1.430
PT1	2.802
PT2	3.241
PT3	2.167
SI1	2.212
SI2	2.241
SI3	1.519

**Table 7:** Path coefficient values.

	Original Sample (O)	Sample Mean (M)	Standard Deviation (STDEV)	T Statistics (|O/STDEV|)	*p* Values
FC → BI	0.128	0.128	0.061	2.087	0.037
PEOU → BI	0.141	0.142	0.061	2.313	0.021
PI → BI	0.173	0.174	0.058	2.954	0.003
PT → BI	0.310	0.307	0.065	4.806	0.000
SI → BI	0.263	0.263	0.067	3.941	0.000

Source: SMART PLS Analysis Result.

**Table 8:** Summary of hypotheses.

**H1**: *Perceived ease of use is positively related to behavioral intention to use fintech services.*	Supported
**H2**: *Social influence is positively related to behavioral intention to use fintech services.*	Supported
**H3**: *Facilitating conditions are positively related to behavioral intention to use fintech services.*	Supported
**H4**: *Personal innovativeness is positively related to behavioral intention to use fintech services.*	Supported
**H5**: *Perceived trust is positively related to behavioral intention to use fintech services.*	Supported

## Data Availability

Not applicable.

## Appendix A. Measurement Constructs of the Variables

**Variables**	**Measurement Constructs**
Behavioral Intention to Adopt Fintech Services (BI)	BI1: I expect to accept fintech services in the future for healthcare payments. BI2: To perform healthcare transactions I predict to use fintech services in future BI3: I will recommend others to use fintech services for healthcare payments.
Perceived Ease of Use (PEOU)	PEOU1: It is easy to use fintech services for healthcare related transactions PEOU2: I think the operation interface of Fintech is friendly and understandable PEOU3: It is easy to have device to use Fintech services PEOU4: I find fintech apps to be flexible to interact with
Social Influence (SI)	SI1: People who are valuable to me feel that I should use Fintech Services for healthcare payments. SI2: People who affect my behavior feel that I should use Fintech Services for healthcare payments. SI3: People whose thoughts I value prefer that I use Fintech Services for healthcare related payments.
Facilitating Conditions (FC)	FC1: I have necessary resources to use Fintech Services FC2: I have the necessary knowledge to use Fintech Services FC3: Fintech Services is compatible with other system that I use
Personal Innovativeness (PI)	PI1: If I heard about new fintech payment options for healthcare payment, I would look for ways to experiment with it. PI2: I like to experiment new fintech services. PI3: In general, I am not hesitant to try out new fintech services
Perceived Trust (PT)	PT1: I trust fintech systems to be reliable. PT2: I trust fintech systems to be secure. PT3: I believe fintech systems are trustworthy

## References

[1] Kim Y., Park Y.J., Choi J. (2016). The Adoption of Mobile Payment Services for “Fintech”. Int. J. Appl. Eng. Res..

[2] Statista (2021). Fintech—Statistics & Facts. https://www.statista.com/statistics/893954/number-fintech-startups-by-region/.

[3] EY Global Financial Services (2021). Global FinTech Adoption Index 2019. https://assets.ey.com/content/dam/ey-sites/ey-com/en_gl/topics/banking-and-capital-markets/ey-global-fintech-adoption-index.pdf.

[4] Rana A. How FinTech is Enhancing Growth in Healthcare Industry. 2021, Cogneesol. https://www.cogneesol.com/blog/fintech-is-enhancing-growth-in-healthcare/#:~:text=Innovations%20in%20Fintech%20Helping%20Healthcare%20Sector&text=Fintech%20companies%20are%20targeting%20the,easy%20%E2%80%93%20single%2Dclick%20option..

[5] BIDA (2022). Healthcare. https://bida.gov.bd/healthcare.

[6] Beckar’s Hospital Review (2017). Top 4 Challenges to Obtain Patients Payment. https://www.beckershospitalreview.com/finance/top-4-challenges-of-obtaining-patient-payments.html.

[7] Keystone Healthcare Studies (2018). What Healthcare And Fintech Have in Common. https://www.healthcarestudies.com/article/what-healthcare-and-fintech-have-in-common/.

[8] Surf (2022). How to Build Fintech Trust with App Users: Top 5 UX Design Practices. https://surf.dev/how-to-build-fintech-trust-with-app-users-top-5-ux-design-practices/.

[9] Mailizar M., Almanthari A., Maulina S. (2021). Examining teachers’ behavioral intention to use E-learning in teaching of mathematics: An extended TAM model. Contemp. Educ. Technol..

[10] Kwateng K.O., Atiemo K.A.O., Appiah C. (2018). Acceptance and use of mobile banking: An application of UTAUT2. J. Enterp. Inf. Manag..

[11] Ali M., Raza S.A., Puah C.H., Amin H. (2019). Consumer acceptance toward takaful in Pakistan: An application of diffusion of innovation theory. Int. J. Emerg. Mark..

[12] Slawinski S. (2021). 5 Reasons Why Fintech Is Important. Global Fintech News. https://globalfintechnews.com/5-reasons-why-fintech-is-important/.

[13] Singh S., Sahni M.M., Kovid R.K. (2020). What drives FinTech adoption? A multi-method evaluation using an adapted technology acceptance model. Manag. Decis..

[14] Ryu H.S. Understanding benefit and risk framework of fintech adoption: Comparison of early adopters and late adopters. Proceedings of the 51st Hawaii International Conference on System Sciences.

[15] Setiawan B., Nugraha D.P., Irawan A., Nathan R.J., Zoltan Z. (2021). User innovativeness and fintech adoption in Indonesia. J. Open Innov. Technol. Mark. Complex..

[16] Chan R., Troshani I., Rao Hill S., Hoffmann A. (2022). Towards an understanding of consumers’ FinTech adoption: The case of Open Banking. Int. J. Bank Mark..

[17] Bureshaid N., Lu K., Sarea A. (2021). Adoption of FinTech Services in the Banking Industry. Applications of Artificial Intelligence in Business, Education and Healthcare.

[18] Davis F.D. (1989). Perceived usefulness, perceived ease of use, and user acceptance of information technology. MIS Q..

[19] Rahmi B.A.K.I., Birgoren B., Aktepe A. (2018). A meta-analysis of factors affecting perceived usefulness and perceived ease of use in the adoption of e-learning systems. Turk. Online J. Distance Educ..

[20] Nangin M.A., Barusi R.G., Wahyoedi S. (2020). The Effects of Perceived Ease of Use, Security, and Promotion on Trust and Its Implications on Fintech Adoption. J. Consum. Sci..

[21] Moslehpour M., Pham V.K., Wong W.K., Bilgiçli İ. (2018). E-purchase intention of Taiwanese consumers: Sustainable mediation of perceived usefulness and perceived ease of use. Sustainability.

[22] Wicaksono A., Maharani A. (2020). The effect of perceived usefulness and perceived ease of use on the technology acceptance model to use an online travel agency. J. Bus. Manag. Rev..

[23] Venkatesh V., Thong J.Y., Xu X. (2012). Consumer acceptance and use of information technology: Extending the unified theory of acceptance and use of technology. MIS Q..

[24] Yusof H., Munir M.F.M.B., Zolkaply Z., Jing C.L., Hao C.Y., Ying D.S., Leong T.K. (2018). Behavioral intention to adopt blockchain technology: Viewpoint of the banking institutions in Malaysia. Int. J. Adv. Sci. Res. Manag..

[25] Prabhakaran S., Vasantha D.S., Sarika P. (2020). Effect of social influence on intention to use the mobile wallet with the mediating effect of promotional benefits. J. Xi’an Univ. Archit. Technol..

[26] Alalwan A.A., Dwivedi Y.K., Rana N.P. (2017). Factors influencing adoption of mobile banking by Jordanian bank customers: Extending UTAUT2 with trust. Int. J. Inf. Manag..

[27] Brown S.A., Venkatesh V. (2005). A model of adoption of technology in the household: A baseline model test and extension incorporating household life cycle. Manag. Inf. Syst. Q..

[28] Venkatesh V., Morris M.G., Davis G.B., Davis F.D. (2003). User acceptance of information technology: Toward a unified view. MIS Q..

[29] Triandis H.C. (1980). Attitudes, values, and interpersonal behaviour. 1979 Nebraska Symposium on Motivation.

[30] Khan I.U., Hameed Z., Khan S.U. (2017). Understanding online banking adoption in a developing country: UTAUT2 with cultural moderators. J. Glob. Inf. Manag. (JGIM).

[31] Widodo M., Irawan M.I., Sukmono R.A. Extending UTAUT2 to explore digital wallet adoption in Indonesia. Proceedings of the 2019 International Conference on Information and Communications Technology (ICOIACT).

[32] Megadewandanu S. Exploring mobile wallet adoption in Indonesia using UTAUT2: An approach from a consumer perspective. Proceedings of the 2016 2nd International Conference on Science and Technology-Computer (ICST).

[33] Marpaung F.K., Dewi R.S., Grace E., Sudirman A., Sugiat M. (2021). Behavioural Stimulus for Using Bank Mestika Mobile Banking Services: UTAUT2 Model Perspective. Gold. Ratio Mark. Appl. Psychol. Bus..

[34] Khazaei H. (2019). The influence of personal innovativeness and price value on intention to use electric vehicles in Malaysia. Eur. Online J. Nat. Soc. Sci..

[35] Turan A., Tunç A.Ö., Zehir C. (2015). A theoretical model proposal: Personal innovativeness and user involvement as antecedents of the unified theory of acceptance and use of technology. Procedia-Soc. Behav. Sci..

[36] Agarwal R., Prasad J. (1998). A conceptual and operational definition of personal innovativeness in the domain of information technology. Inf. Syst. Res..

[37] Lu J. (2014). Are personal innovativeness and social influence critical to continue with mobile commerce?. Internet Res..

[38] Cao Q., Niu X. (2019). Integrating context-awareness and UTAUT to explain Alipay user adoption. Int. J. Ind. Ergon..

[39] Suebtimrat P., Vonguai R. (2021). An Investigation of Behavioral Intention Towards QR Code Payment in Bangkok, Thailand. J. Asian Financ. Econ. Bus..

[40] Damghanian H., Zarei A., Siahsarani Kojuri M.A. (2016). Impact of perceived security on trust, perceived risk, and acceptance of online banking in Iran. J. Internet Commer..

[41] Kim D.J., Ferrin D.L., Rao H.R. (2008). A trust-based consumer decision-making model in electronic commerce: The role of trust, perceived risk, and their antecedents. Decis. Support Syst..

[42] Stewart H., Jürjens J. (2018). Data security and consumer trust in FinTech innovation in Germany. Inf. Comput. Secur..

[43] Arkorful V.E., Lugu B.K., Hammond A., Basiru I., Afriyie F.A., Mohajan B. (2021). Examining quality, value, satisfaction and trust dimensions: An empirical lens to understand health insurance systems actual usage. Public Organ. Rev..

[44] Fishbein M., Ajzen I. (1975). Belief, Attitude, Intention, and Behavior: An Introduction to Theory and Research.

[45] Ajzen I. (2012). The theory of planned behavior. Handbook of Theories of Social Psychology.

[46] Al Nawayseh M.K. (2020). Fintech in COVID-19 and beyond: What factors are affecting customers’ choice of fintech applications?. J. Open Innov. Technol. Mark. Complex..

[47] Hassan M.S., Islam M.A., Sobhani F.A., Nasir H., Mahmud I., Zahra F.T. (2022). Drivers Influencing the Adoption Intention towards Mobile Fintech Services: A Study on the Emerging Bangladesh Market. Information.

[48] Wilson N. (2019). The impact of perceived usefulness and perceived ease-of-use toward repurchase intention in the Indonesian e-commerce industry. J. Manaj. Indones..

[49] Mokhtar S.A., Katan H., Hidayat-ur-Rehman I. (2018). Instructors’ behavioural intention to use learning management system: An integrated TAM perspective. TEM J..

[50] Sun Y., Gao F. (2020). An investigation of the influence of intrinsic motivation on students’ intention to use mobile devices in language learning. Educ. Technol. Res. Dev..

[51] Patel K.J., Patel H.J. (2018). Adoption of internet banking services in Gujarat: An extension of TAM with perceived security and social influence. Int. J. Bank Mark..

[52] Zhou M., Huang J., Wu K., Huang X., Kong N., Campy K.S. (2021). Characterizing Chinese consumers’ intention to use live e-commerce shopping. Technol. Soc..

[53] Blaise R., Halloran M., Muchnick M. (2018). Mobile commerce competitive advantage: A quantitative study of variables that predict m-commerce purchase intentions. J. Internet Commer..

[54] Simarmata M.T., Hia I.J. (2020). The role of personal innovativeness on the behavioral intention of Information Technology. J. Econ. Bus..

[55] Grandison T., Sloman M. (2000). A survey of trust in internet applications. IEEE Commun. Surv. Tutor..

[56] Wang S.W., Ngamsiriudom W., Hsieh C.H. (2015). Trust disposition, trust antecedents, trust, and behavioral intention. Serv. Ind. J..

[57] Mahwadha W.I. (2019). The behavioral intention of young consumers towards E-wallEt adoption: An empirical study among Indonesian users. Russ. J. Agric. Socio-Econ. Sci..

[58] Barclay D., Higgins C., Thompson R. (1995). The partial least squares (PLS) approach to casual modelling: Personal computer adoption and use as an Illustration. Technol. Stud..

[59] Chen W.-C., Chen C.-W., Chen W.-K. (2019). Drivers of Mobile Payment Acceptance in China: An Empirical Investigation. Information.

[60] Hu Z., Ding S., Li S., Chen L., Yang S. (2019). Adoption intention of fintech services for bank users: An empirical examination with an extended technology acceptance model. Symmetry.

[61] Patil P., Tamilmani K., Rana N.P., Raghavan V. (2020). Understanding consumer adoption of mobile payment in India: Extending Meta-UTAUT model with personal innovativeness, anxiety, trust, and grievance redressal. Int. J. Inf. Manag..

[62] Chin A.G., Harris M.A., Brookshire R. (2020). An Empirical Investigation of Intent to Adopt Mobile Payment Systems Using a Trust-based Extended Valence Framework. Inf. Syst. Front..

[63] Jiang Q., Chen J., Wu Y., Gu C., Sun J. (2022). A Study of Factors Influencing the Continuance Intention to the Usage of Augmented Reality in Museums. Systems.

[64] Eutsler J., Lang B. (2015). Rating scales in accounting research: The impact of scale points and labels. Behav. Res. Account..

[65] Hair J.F., Hult G.T.M., Ringle C.M., Sarstedt M., Thiele K.O. (2017). Mirror on the Wall: A Comparative Evaluation of Composite-based Structural Equation Modeling Methods. J. Acad. Mark. Sci..

[66] Hair J.F., Ringle C.M., Sarstedt M. (2011). PLS-SEM: Indeed a Silver Bullet. J. Mark. Theory Pract..

[67] Hair J.F., Risher J.J., Sarstedt M., Ringle C.M. (2019). When to use and how to report the results of PLS-SEM. Eur. Bus. Rev..

[68] Fornell C., Larcker D.F. (1981). Evaluating structural equation models with unobservable variables and measurement error. J. Mark. Res..

[69] Henseler J., Ringle C.M., Sarstedt M. (2015). A new criterion for assessing discriminant validity in variance-based structural equation modelling. J. Acad. Mark. Sci..

[70] The World Bank (2022). Current Health Expenditure (% of GDP)—Bangladesh. https://data.worldbank.org/indicator/SH.XPD.CHEX.GD.ZS?locations=BD.

[71] Macrotrends Bangladesh Healthcare Spending 2000–2022. https://www.macrotrends.net/countries/BGD/bangladesh/healthcare-spending#:~:text=Bangladesh%20healthcare%20spending%20for%202019,a%205.24%25%20increase%20from%202015.

[72] Statista (2022). Digital Markets Fintech Bangladesh. https://www.statista.com/outlook/dmo/fintech/bangladesh.

